# Nano soil improvement technique using cement

**DOI:** 10.1038/s41598-023-37918-z

**Published:** 2023-07-03

**Authors:** Hamed Niroumand, Lech Balachowski, Reza Parviz

**Affiliations:** 1 Department of Civil Engineering, Faculty of Engineering, Buein Zahra Technical University (IKIU-BZ), Qazvin, Iran; 2 Department of Geotechnical and Hydraulic Engineering, Faculty of Civil and Environmental Engineering, Gdansk University of Technology, Gdansk, Poland

**Keywords:** Civil engineering, Chemistry

## Abstract

Nano soil-improvement is an innovative idea in geotechnical engineering. Nanomaterials are among the newest additives that improve soil properties. Herein, laboratory tests, such as unconfined compressive strength, direct shear test, and initial tests, were conducted to investigate the geotechnical properties of Kelachay clay with micro- and nanosized cement to evaluate its particles in untreated soil and observe changes in the behavioral properties of treated soil compared to those of untreated soil. Scanning electron microscopy and X-ray fluorescence images were analyzed before and after the grinding process to determine the nature of the studied particles. Furthermore, effects of time and nanocement content (0%, 1%, 3%, 5%, and 7%) on curing performance were evaluated. The optimum percentage of nano-cement was found to be 7%, which increased the unconfined compressive strength by up to 29 times and reduced the strain at rupture by 74% compared to the untreated soil. The results showed that nano-cement significantly improved the strength and stiffness of the soil–cement mixture by forming calcium silicate hydrate (C–S–H) gel that filled the pores and bonded the soil particles. Nano-cement also acted as a nucleation site for more C–S–H growth, enhancing the durability and strength of the mixture.

## Introduction

Soil improvement techniques are important in geotechnical engineering. Soil stabilization is a method for improving soil properties. Barman and Dash described various methods for expansive soil stabilization using chemical additives^1^. According to their analysis, such chemical methods exhibited various gaps such as hydration, cation exchange, flocculation, and pozzolanic reactions. Furthermore, various factors such as additive type, additive content, soil type, soil mineralogy, curing period, curing temperature, delay in compaction, pH of soil matrix, and molding water content affect soil stabilization performance.

Although traditional soil stabilization methods have improved various parameters of cohesive soil, modern methods are required for improving traditional techniques for soil stabilization in various geotechnical projects. A common method to improve or modify soil properties is to change the properties of the soil at the site^2^. Since its inception in the 1990s, nanoscience has been the buzzword. Nanoscience refers to any technology that operates at the nanoscale or molecular level. Nanotechnology involves the production and use of physical, chemical, and biological systems at scales ranging from atomic or molecular to submicron level as well as the incorporation of the resulting nanostructure into larger systems. Taha investigated the geotechnical properties of soil mixed with nanosoil particles^3^. Experiments were conducted to investigate the geotechnical properties of natural soil mixed with nanosoil. Results showed that the values of liquidity and plasticity limits increased after the addition of the nanosoil. Further, the compressive strength of soil containing 1% nanosoil was approximately double that of natural soil. Moreover, the presence of even a little amount of nanosoil was found to considerably improve the soil properties.

Tah and Taha conducted several studies on the effect of nanomaterials as nanoparticles on the creep and swelling behavior of soil^4^. Four types of soil were combined in varying proportions with three types of nanomaterials for soil experiments. Finally, it was found that the addition of additives decreased the optimal percentage of strain owing to creep and swelling. Further, the nanomaterials reduced dry surface cracking during compaction without reducing hydraulic conductivity (permeability). Soil and nanomaterial mixtures improved the soil properties, such as compaction, volumetric strain resulting from creep and swelling, and crack intensity factor (CIF). Although the addition of nanoclay improves many soil properties, it has a considerable impact on Atterberg limits, which is a negative aspect of nanoclay properties. Improvement of the creep and swelling strains of the soil achieved using nanocopper is much higher than that achieved using nanoaluminum owing to the higher compaction of copper particles.

Chasabkolaei et al. described numerous applications of nanoparticles in various fields of architecture and civil engineering, particularly in the field of soil stabilization^5^. Several nanomaterials have been employed to stabilize soil. Baziar et al*.* investigated the role of clay nanoparticles as additives, demonstrating the hydraulic conductivity of cohesive soil^6^.

Nanoscale zerovalent iron (nZVI), titanium dioxide (TiO_2_), zinc oxide (ZnO), multiwalled carbon nanotubes (MWCNTs), fullerenes, bimetallic nanoparticles, and stabilized nanoparticles are the most widely utilized nanoparticles to improve soil properties^7,8^. Owing to their sizes and properties, nanomaterials have considerably expanded surface areas and sorption sites, making them excellent absorbents^7,8^. Soil improvement is a traditional strategy to meet soil requirements for various applications^9^. Traditionally, cement and mineral additives such as fly ash, silica fume, and rice husk ash were used to improve soil properties^9^.

Soil and rock minerals contain nanomaterials such as halloysite, sepiolite, hematite, allophane, smectite, imogolite, palygorskite, and goethite. Owing to the large specific surface area of soil particles containing organic matter and clay minerals, nanoparticles can affect the physical and chemical properties and the microstructure of soil^9^. Jahanian et al. investigated the effect of two powdered materials, Portland cement and nanocement, in improving soil properties^10^. They evaluated the geotechnical aspects of these powders in cohesive soils. A uniaxial compressive strength test was performed on the specimens with curing times of 7, 14, 28, and 42 days. Results showed that the increase in the uniaxial compressive strength of the specimens was approximately 10 and 12 times greater than that of the prototype.

Eyo et al. investigated the performance of clay with RoadCem (RC) as nanoadditives^11^. They evaluated various geotechnical properties such as unconfined compression tests and one-dimensional oedometer swelling. Treated clay with RC reduced porosity due to nanosize of particles. This stabilized clay could improve clay properties.

Niu et al. provided a review of the properties of cement-based materials containing nanoclay and calcined nanoclay^12^. They reported that nanoclay, a type of nanoadditive, exhibited positive effects on improving various properties of cement-based materials. Garg et al. emphasized the effect of partial substitution of cement/addition of various nanoadditives on various aspects of strength and structure analysis^13^. They evaluated several additives/supplementary materials as partial substitutions of cement. Selvakumar et al. evaluated the influence of silica nanoparticles and sodium silicate on clayey soil's strength parameters^14^. Kulanthaivel et al. investigated how nano-silica, fibers, bio-cemented egg shell, other nano-materials, and white cement affect clay and sandy soils^15–18^.

Liu et al. evaluated the effects of mining operations on nanoadditive management using artificial intelligence models of an artificial neural network (ANN) and extreme learning machine (ELM)^19^. Cement is one of the most widely used materials in the construction industry. Sivabalaselvamani et al. evaluated the role of machine learning and related algorithms on soil stabilization with ceramic powder^20^.

Kannan et al. evaluated the role of nanocalcium carbonate (NCC) in treating low-plasticity organic soil^21^. They described various geotechnical aspects of NCC in soil stabilization. According to their research, various geotechnical tests such as the plasticity index, compaction, unconfined compressive strength (UCS), compressibility and permeability characteristics of the 0.2%, 0.4%, 0.6%, and 0.8% NCC-stabilized soil, and untreated soil were evaluated. They proposed that the NCC is a viable additive for soil improvement techniques. An optimum dosage of 0.4% NCC additive is suggested for soil stabilization.

Recently, nano-additives materials with excellent mechanical properties have been widely used for soil stabilization.

According to a literature review, no research has been conducted on preparing nanoparticles without the addition of chemicals using mechanical methods that are low-cost and energy-efficient. This research examined the effect of soil stabilization by micro- and nano-cement via the suspension method, as there is a lack of research on the effects of the suspension base of nanocement on clay. This research evaluated micro- and nano-cements as an innovative technique of soil stabilization for soil improvement. This research used soluble nanoparticles to stabilize and improve the soil properties, considering the economic justification and optimal use of cement nanoparticles. To the best of our knowledge, this has not been done previously.

## Materials and methods

### Soil specimen

**Figure 1:**
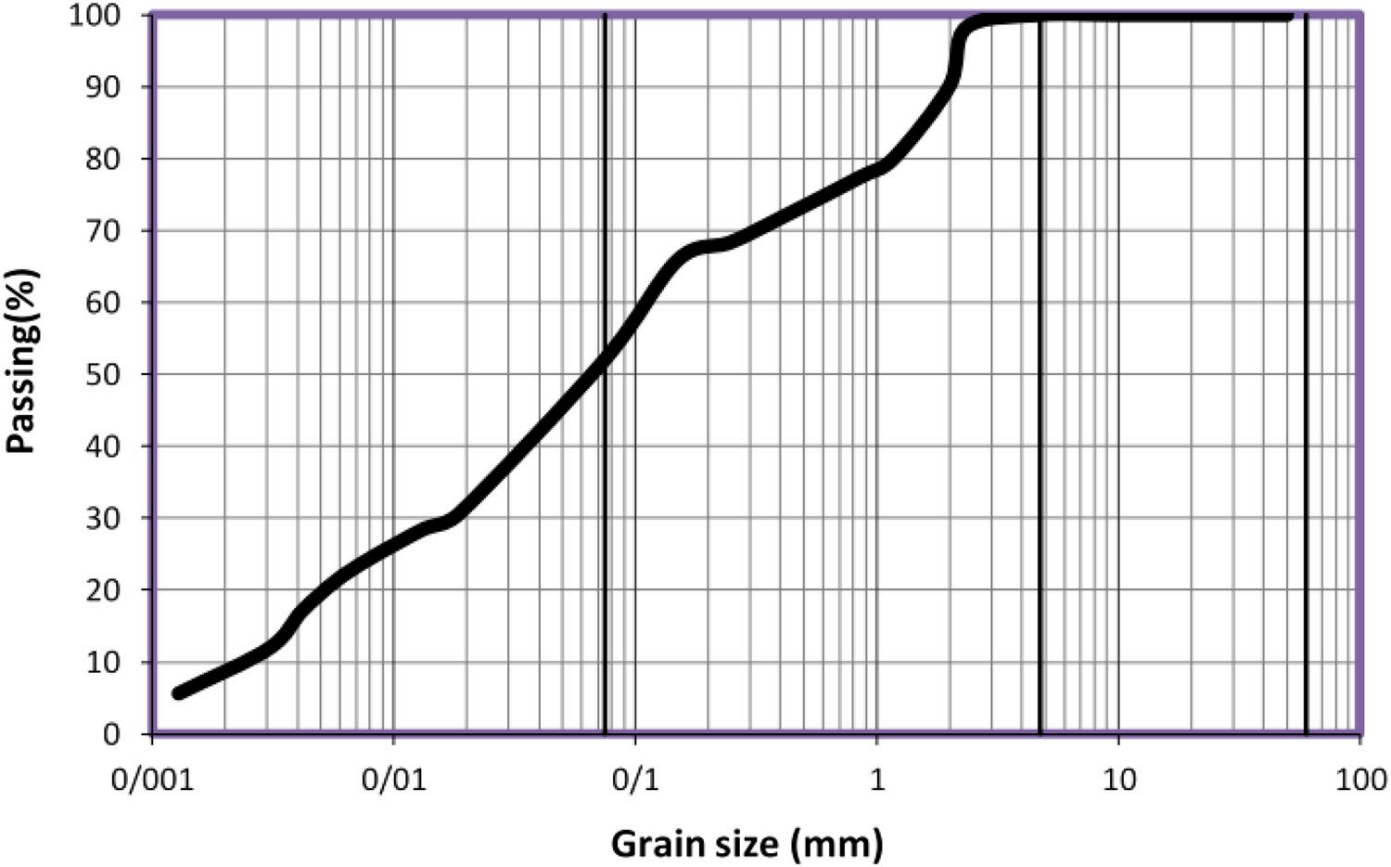
Curve of the studied soil granulation.

Herein, the clay soil of Kelachay, a region located in the north of Iran (Gilan province, Iran), was used. Kelachay city is located on the Caspian Sea. Sieve and hydrometer granulation tests were performed according to the standard ASTM D422^22^, as shown in Fig. 1.

According to the standard ASTM D431^23^, liquidity and plasticity limit tests were performed. Table 1 presents the results. According to the results of granulation and Atterberg limits, the soil specimen was classified as the CL based on the unified soil classification system (USCS).

**Table 1:** Index properties of soil based on sieve and hydrometer granulation tests in Fig. 1.

Parameter	The amount
LL (%)	34
PL (%)	20
PI (%)	14
USCS classification	CL
Specific gravity (Gs)	2.65
Optimum moisture content (%)	17.3
Maximum dry unit weight (kN/m^3^)	18.3

**Figure 2:**
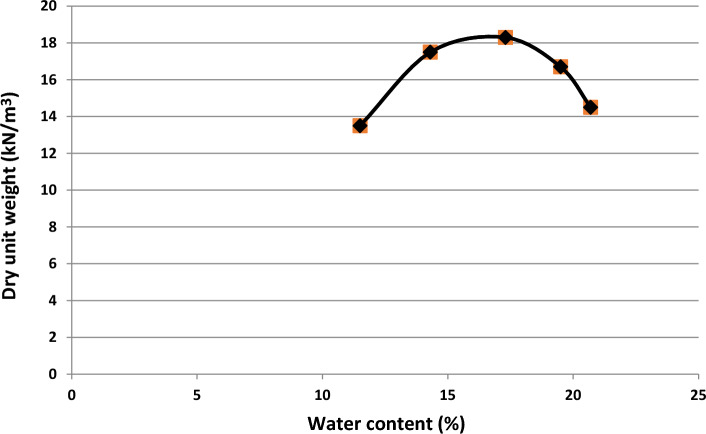
Compaction curve of the soil specimen.

The corrected compaction test was performed according to the standard ASTM D1557^24^, and the maximum specific dry weight of the soil specimen is shown in Fig. 2.

### Specifications of the materials

#### Cement and nanocement

Cement is a type of construction material. Generally, it is used in mortar and concrete. Cement is a form of soil stabilization additive. Powdered alumina, silica, lime, iron oxide, and magnesium oxide were burned in a kiln and pulverized to produce cement. Abi Rekha et al*.* concluded that when the particle size is ~ 10^−9^ m, the chemical activity of materials considerably increases, increasing the ductility of materials and realizing new and valuable properties^25^.

Owing to the lack of the use of chemicals at purification stages, nanocement is considered a suitable product. The existing design uses a “top–down” approach to obtain the nanocement suspension.

The particle size of primary powder, degree of purity, and shape of material particles determine the quality of the material. Herein, the cement particles were microscopic and spherical. Nanostabilization is a type of nanoground improvement. Nanomaterials can be used in dry or soluble forms for soil stabilization; however, the performance of soluble nanomaterials is superior to that of powder particles. As water is used as a lubricant in soil compaction and according to earlier geotechnical studies, water is used as a solvent herein.

### Preparation of the specimens for experimental tests

To examine the given soil specimen using a stabilizer, two categories of tests were considered: soil mechanics tests and tests to investigate the soil structure, effect of structural resistance in terms of structural stabilization of soil in geotechnical projects, design criteria, and rammed soil structures. Soil mechanics tests were performed to observe the effect of soil stabilization, including the two types of unconfined compressive strength (single-axis) and direct shear conditions.

The soil specimen prepared with the treated material differs from the soil specimen prepared with the untreated material. A suspension with a certain proportion is used instead of water in the treated specimen (optimal moisture). The compaction of the tested soil specimens is an important parameter in preparing soil specimens.

For the stabilization of the soil specimen and the investigation of the effectiveness of nanomaterials as the stabilizers of soil structures and replacement of cement, different nanocement contents of 0%, 1%, 3%, 5%, and 7% were applied. Given that cement predominates as a stabilizer in soil structures and that hydration results from the reaction of cement and water, processing times of 28 and 90 days are frequently applied; therefore, these processing times were also considered herein. However, 1- and 7-day intervals were evaluated to determine the effect of varied processing times on various soil specimens treated with nanomaterials. The specimens were placed in a vacuum bag to prevent their evaporation during processing.

#### UCS test

**Figure 3:**
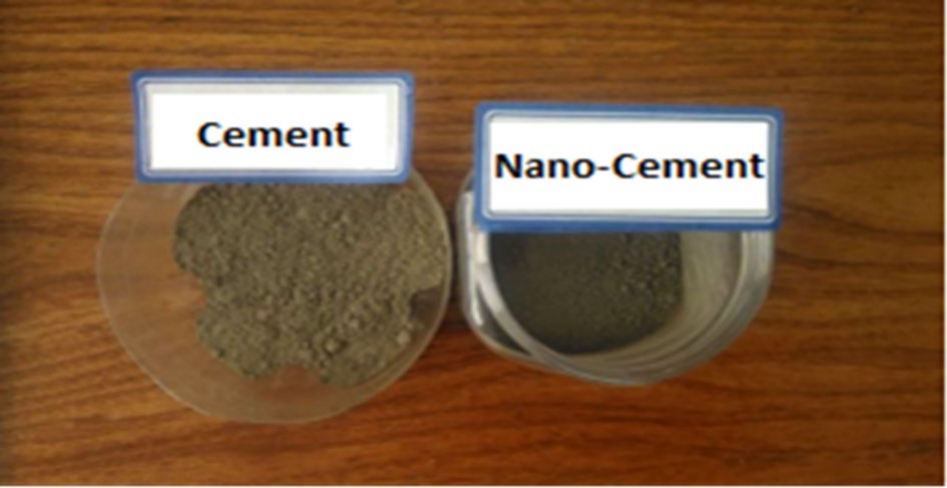
Cement and nanocement samples.

**Figure 4:**
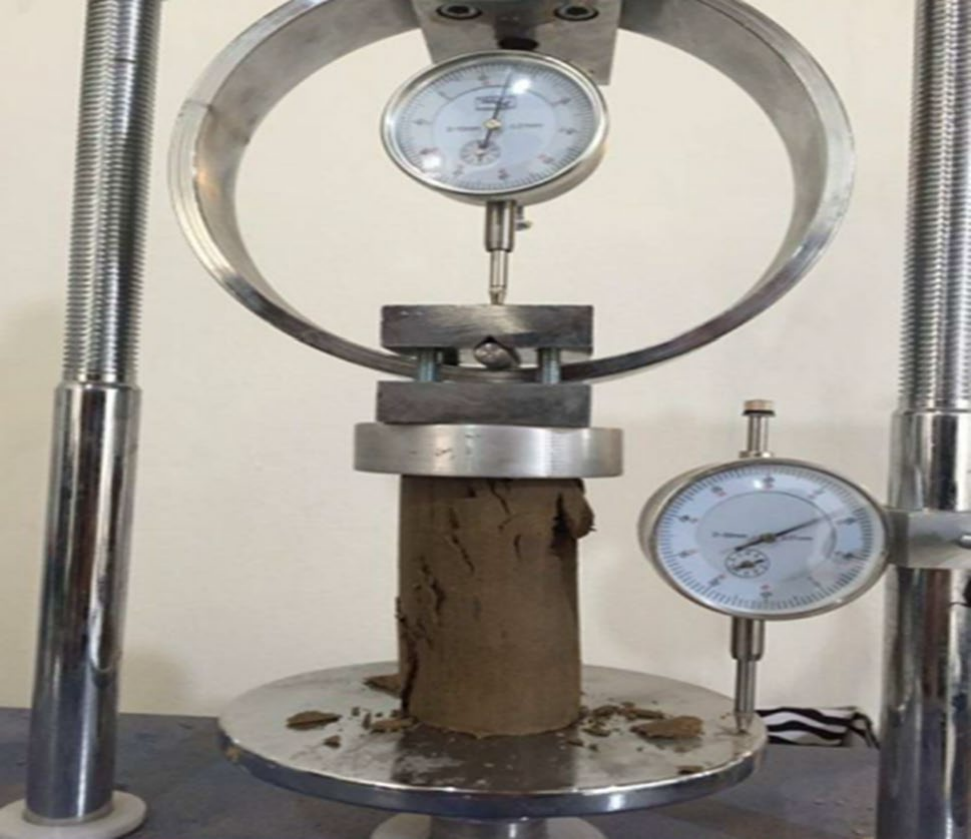
Specimen image after the UCS test.

The unconfined compression tests (q_u_) were conducted utilizing the standard ASTM D2166^26^, which is applicable for adhesive soils. Therefore, the nanocement suspension with various formulation of 1%, 3%, 5%, and 7% of the studied specimens should be prepared based on moisture, optimal moisture percentage, and maximum dry weight obtained from the corrected compaction curve and then added to the soil in accordance with the volume and format of the unconfined compression test at various processing times of 1, 7, 28, and 90 days. The specimen was stored in nylon plastic to preserve the optimal level of moisture. Figure 3 depicts the image of cement and nanocement samples, whereas Fig. 4 depicts an experimental sample.

#### Direct shear test

**Figure 5:**
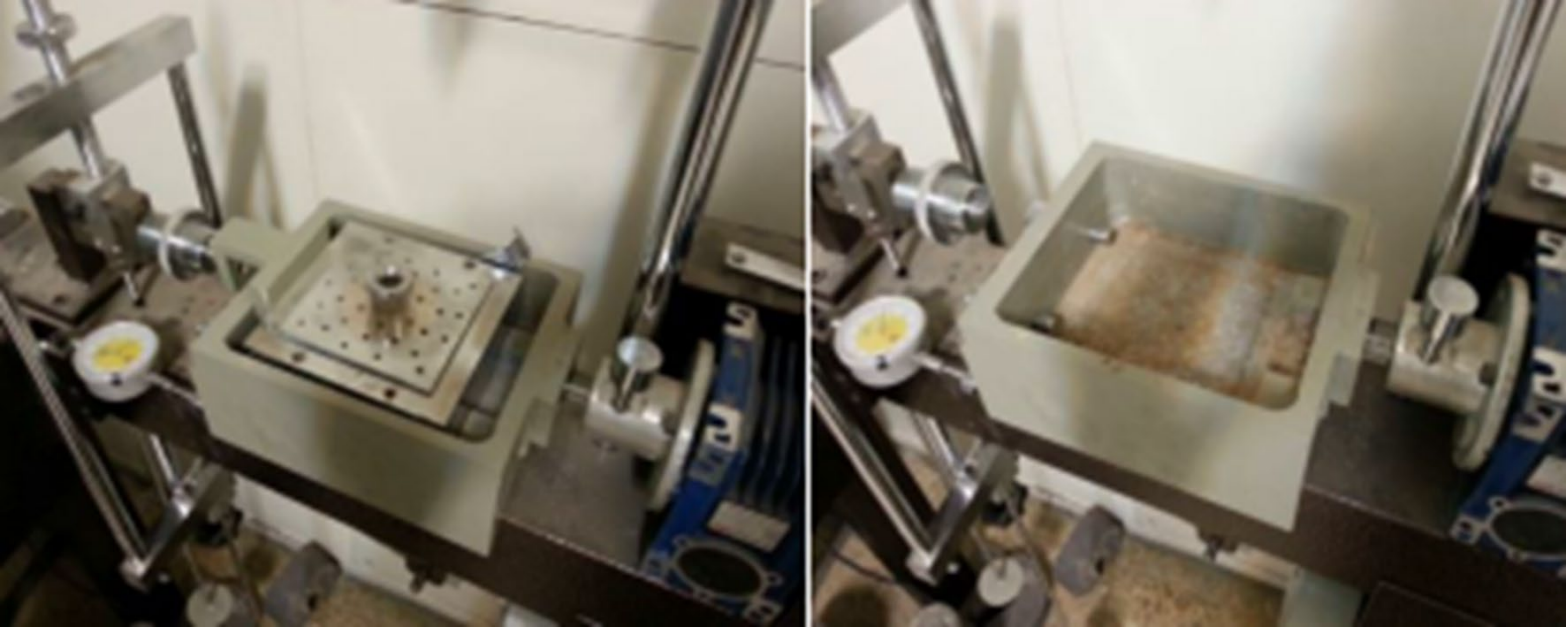
Direct shear test device used herein.

Direct shear test is a form of a laboratory soil test. This test was conducted in accordance with standard ASTM D3080^24^ under three stresses of 50, 100, and 150 kPa on soil specimens treated at 1, 7, 28, and 90 days using 0%, 1%, 3%, 5%, and 7% nanocement. Figure 5 shows the used direct shear test device.

## Results and discussions

### Unconfined compressive strength of untreated soil

**Figure 6:**
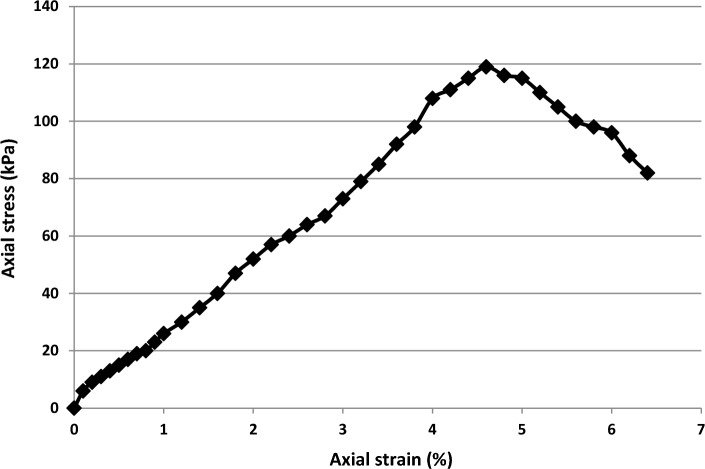
Stress–strain curve of the soil specimen under dry conditions.

Figure 6 shows the stress–strain curve obtained from the unconfined compressive strength test of the soil sample. The maximum stress (119 kPa) in this curve indicated the unconfined compressive strength under dry conditions. Additionally, the breaking strain was 4.6% and the modulus of elasticity (E_50_) was 2.49 MPa, showing that the soil specimen is exceptionally soft. Initially, the curve exhibited an upward trend until it reached a steady state of deformation.

### Effect of cement and nanocement on UCS of soil

**Figure 7:**
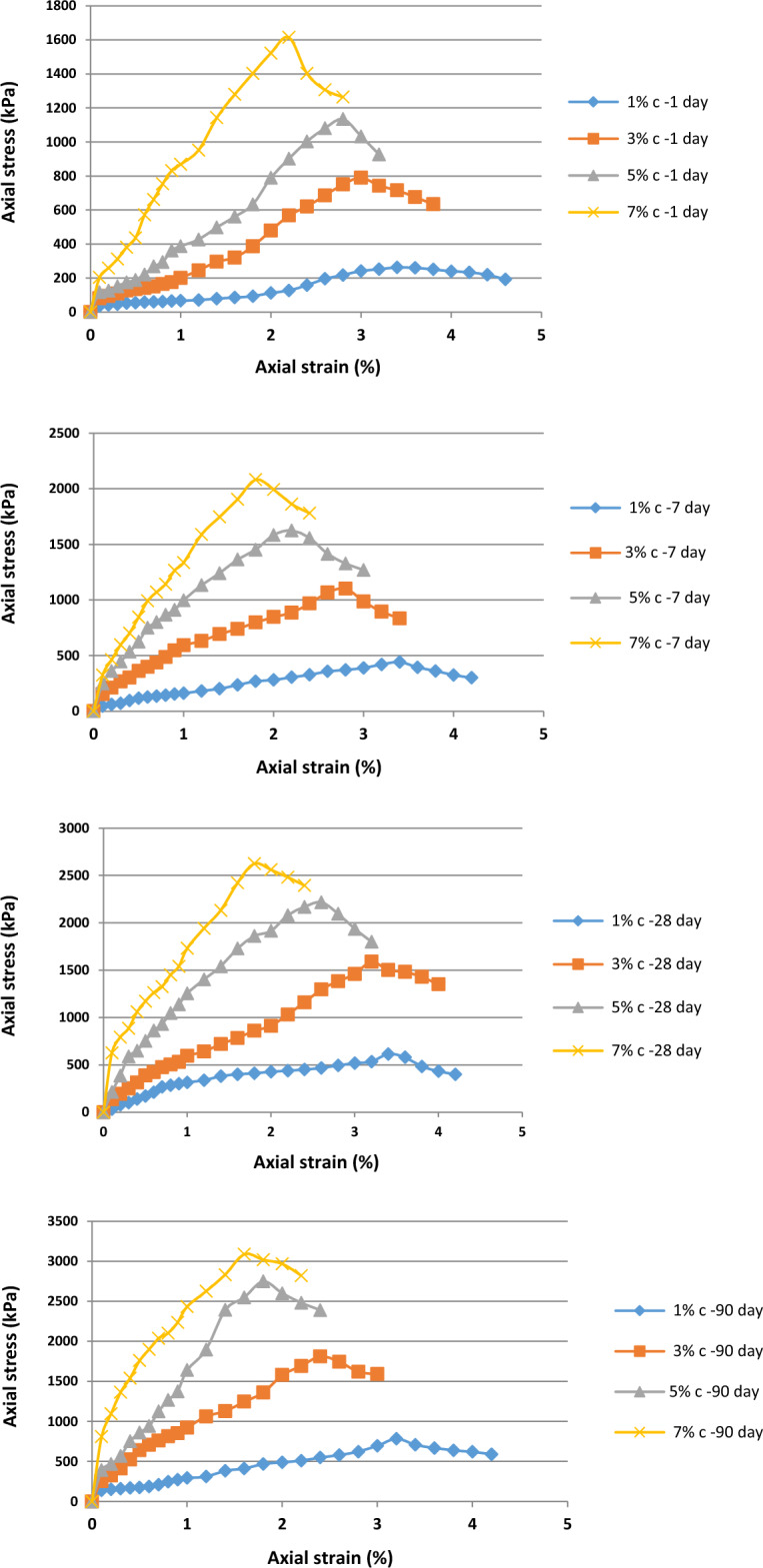
Stress–strain curve of the treated soil specimens with 1%, 3%, 5%, and 7% of cement for curing times of 1, 7, 28, and 90 days.

Figure 7 shows the stress–strain curve obtained from the unconfined compressive strength test for cement used as a soil additive. Owing to its cementation capabilities, cement is used as an independent soil material and can be used to stabilize the soil. Figure 7 shows the stress–strain curve of soil specimens treated with four different percentages of cement particles (1%, 3%, 5%, and 7%) and processing times of 1, 7, 28, and 90 days under dry conditions. Increasing the processing time raised the unconfined, single-axis compressive resistance of the soil specimens.

**Figure 8:**
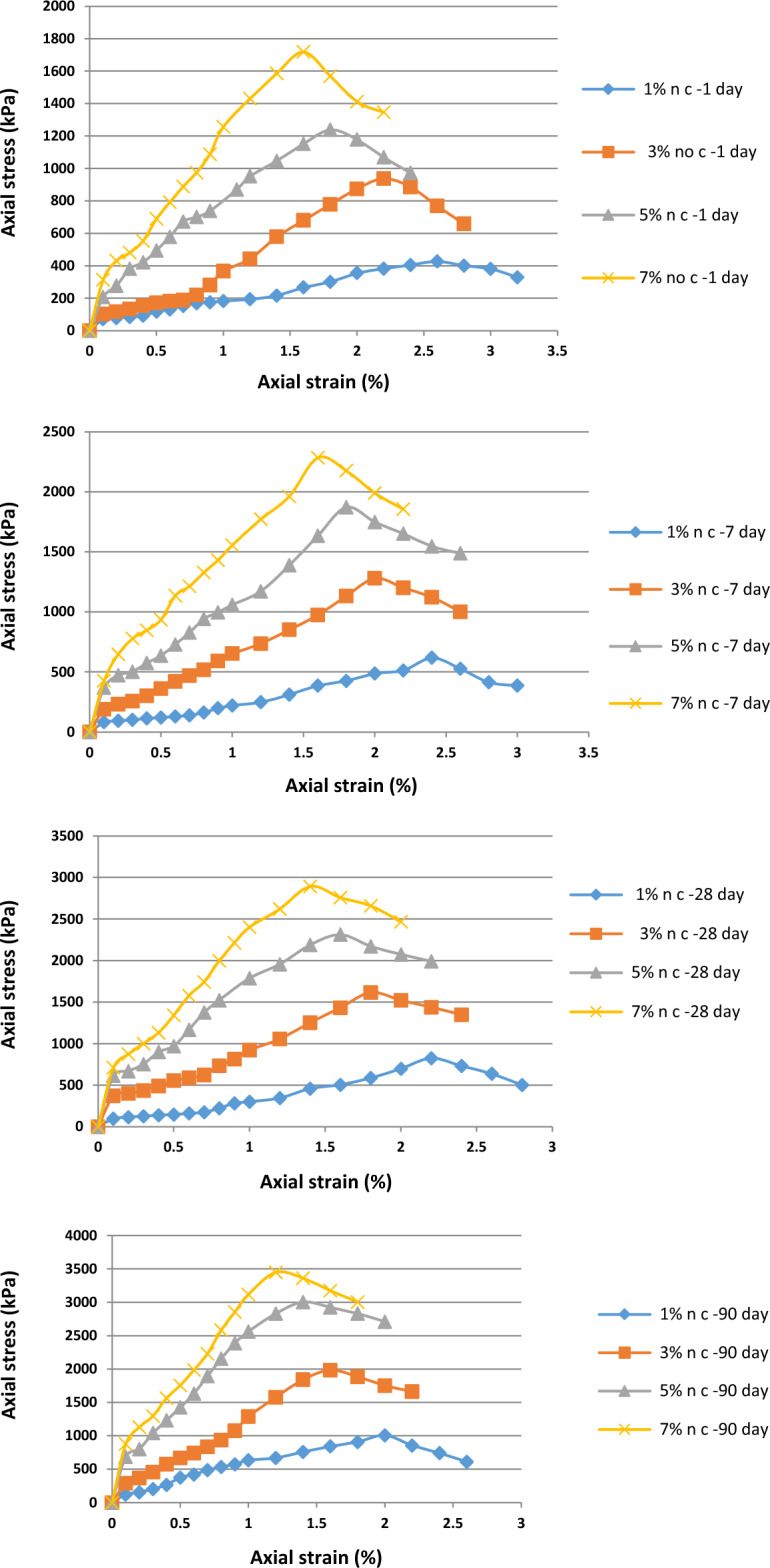
Stress–strain curve of the treated specimens with 1%, 3%, 5%, and 7% of nanocement for curing times of 1, 7, 28, and 90 days.

Figure 8 shows the stress–strain curve obtained from the unconfined compressive strength test for cement used as an independent material in the soil. Nanocement was added to the soil in the percentage range of 1%, 3%, 5%, and 7% by weight and curing times of 1, 7, 28, and 90 days.

**Figure 9:**
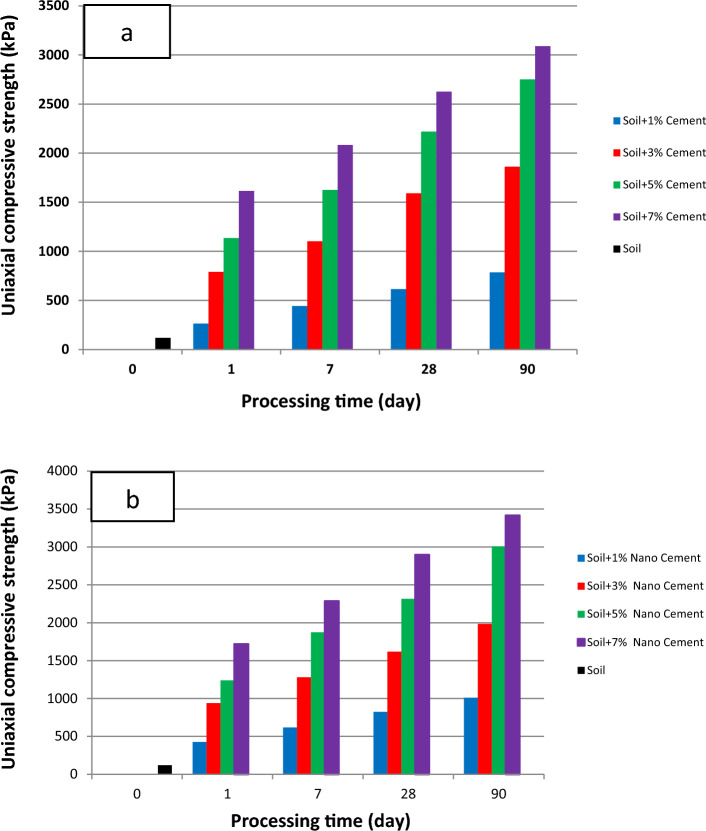
Effect of curing time on unconfined compressive strength of soil specimens with four different percentages of nanocement particles.

Figure 9 shows the effect of curing time on the unconfined compressive strength of soil specimens with the four different percentages of nanocement particles processed for 1, 7, 28, and 90 days under dry conditions. Figure 9 shows that unenclosed resistance to the base clay soil and the soil specimen treated with cement was increased by the addition of 7% of nanoparticles. Additionally, the unconfined single-axis compressive resistance of the soil specimens was enhanced by extending the curing time. Soil behavior becomes more brittle and the level of strain at the moment of breaking decreases with increasing processing time owing to the completion of the cementation reaction and hardening of the soil.

Figure 9 shows the results regarding the effect of processing time on the specimens. During the initial 1–7 days of processing time, the slope of the curve was small and increased until the 90th day of processing, indicating the formation of more bonds between the particles up until the 90th day of processing. Increasing the processing time to increase the unconfined compression strength is an important factor to observe. The compressive strength incrementally increased in the 7-day specimen compared to the 1-day specimen, and this tendency accelerated during the 90-day period. Gradually, cementation chemical reactions between the cement and soil specimen occur during the formation of a new structure. This is owing to the reactions that primarily depend on the physicochemical activity of the clay soil particles, and the goal is to replace a portion of the soil with nanocement containing sufficient cementation additives to perform the cementation chemical reaction in the soil mixture for both short- and long-term reactions occurring in it.

**Figure 10:**
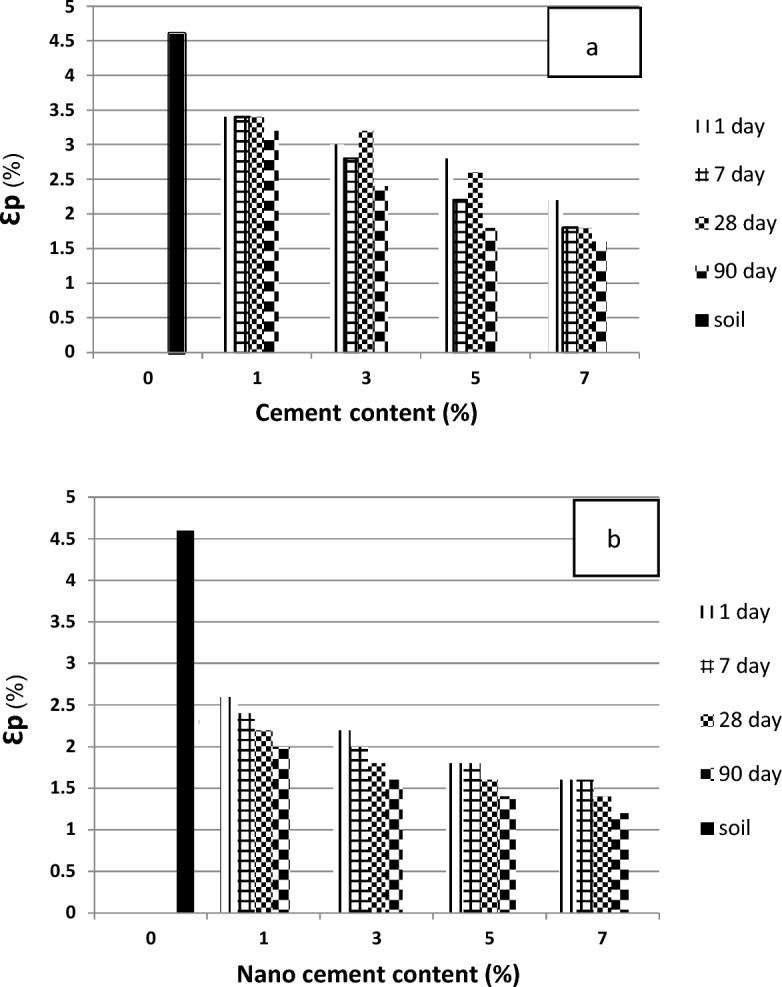
Strain values in the soil specimens untreated and treated using different percentages of (**a**) cement and (**b**) nanocement in various curing times.

Figure 10 shows the breaking strain of the soil specimens treated with various percentages of nanocement and nanocement suspension at the time of processing. The maximum breaking strain was reached by varying the nanocement concentration. Our results regarding the stabilization of the soil specimen with 1% nanocement suspension after 90 days of curing time showed that the breaking strain of the treated soil was ~ 2%, whereas it was ~ 1.2% for the soil treated with 7% nanocement suspension, indicating the dependence of breaking strain changes on nanoparticle concentration.

Table 2 shows the effect of percentages of cement and nanocement suspension during various curing durations on the elastic modulus. Our results showed that the addition of 1%, 3%, 5%, and 7% nanocement suspensions improved the elastic modulus. As shown in Table 2, the modulus of elasticity of soil specimens treated with nanocement exceeded that of the untreated soil.

**Table 2:** Effect of the percentages of cement and nanocement suspension during various curing times on the elastic modulus.

Specimens	${E}_{50}$ (Mpa)								
	1 day	7 days	28 days	90 days	–	1 day	7 days	28 days	90 days
0% Cement	2.49				0% Nano	2.49			
1% Cement	5.93	14.63	32.37	26.39	1% Nano	15.34	22.23	30.15	68.08
3% Cement	21.82	60.55	53.72	93.69	3% Nano	29.45	65.16	92.29	118.37
5% Cement	35.25	112.07	124.88	153.17	5% Nano	96.34	127.96	195.11	277.21
7% Cement	91.76	157.8	191.68	353.58	7% Nano	129.25	184.87	266.48	378.79

### Direct shear test of untreated soil specimen

**Figure 11:**
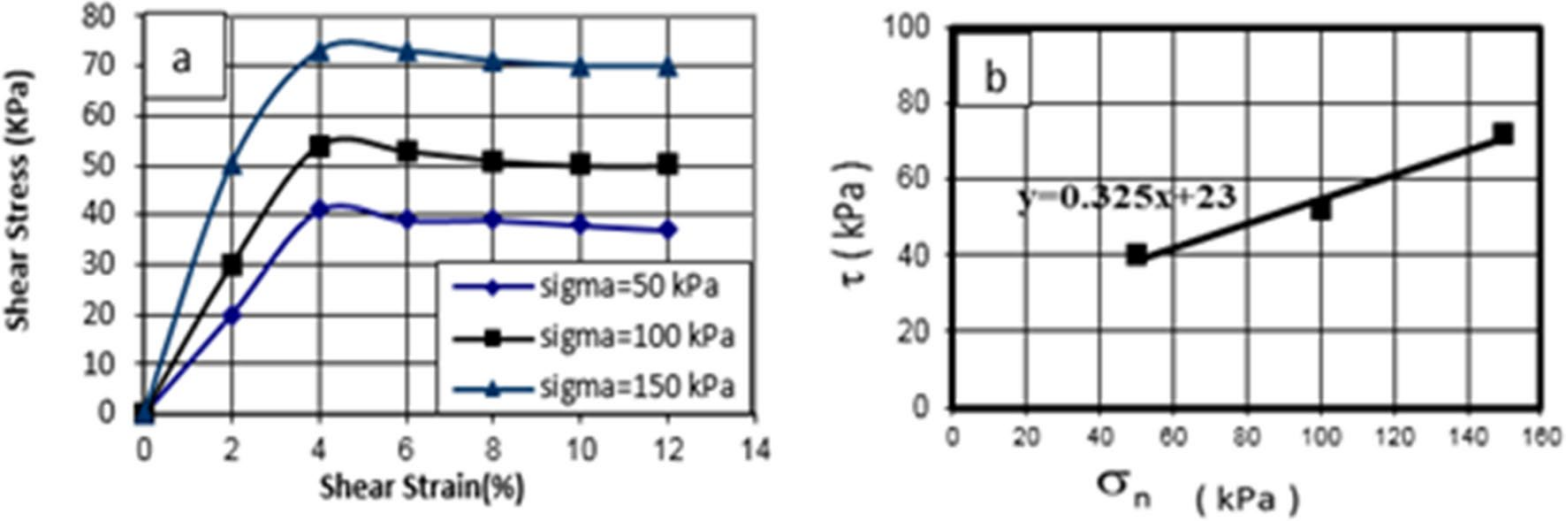
Shear stress–strain curve of the soil specimen under untreated conditions in 28 days curing times.

As shown in Fig. 6, the shear stress–strain behavior of the soil specimen was analyzed under three stresses of 50, 100, and 150 kPa. According to Fig. 11, the maximum shear stress was reached by increasing the shear strain, and then the material ruptured. Shear stress values of 41, 54, and 73 kPa were found for the soil specimen under related stresses of 50, 100, and 150 kPa, respectively.

### Direct shear test of treated soil specimen using cement and nanocement

**Figure 12:**
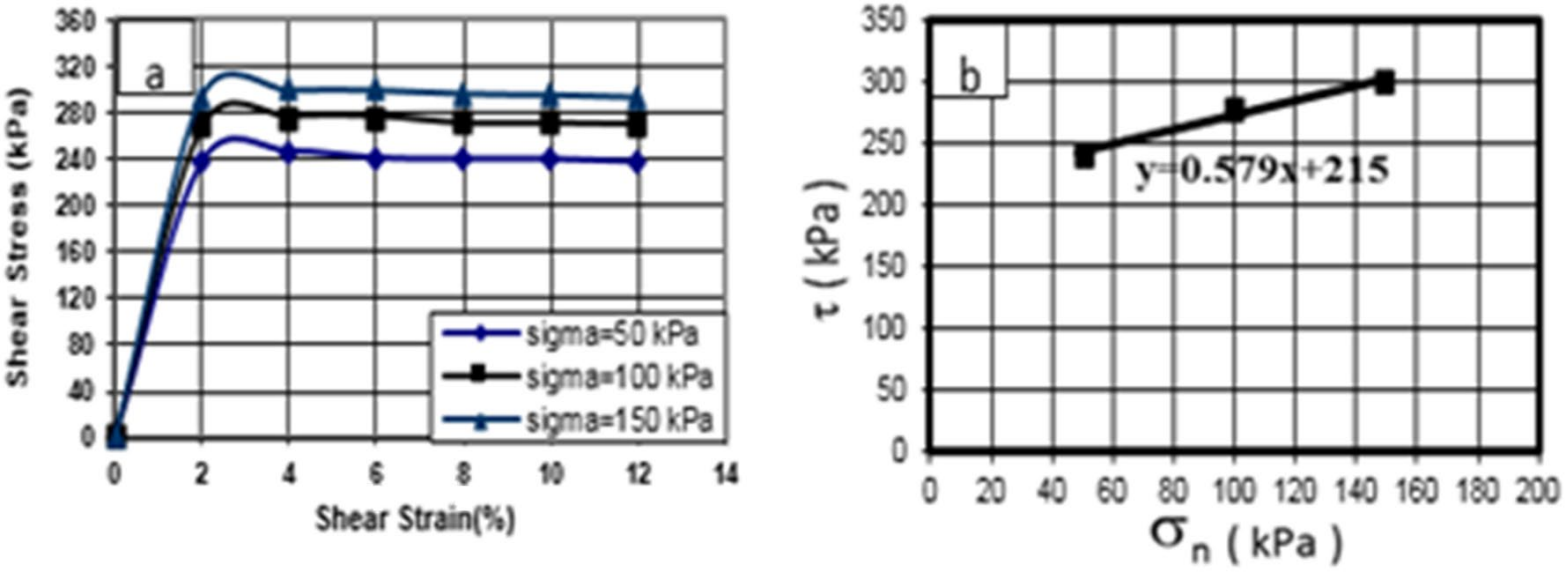
Shear stress–strain curve of the soil specimen under cement-stabilized conditions in curing times of 28 days.

**Figure 13:**
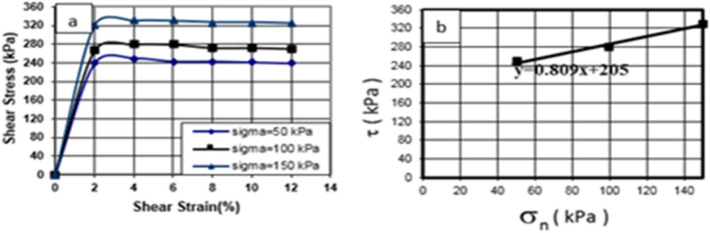
Shear stress–strain curve of the soil specimen under nanocement-stabilized conditions in curing times of 28 days.

The shear stress–strain behavior of the soil specimen with 7% of cement was initially studied under three stresses of 50, 100, and 150 kPa, as shown in Figs. 7 and 8. According to Figs. 12 and 13, shear stress reached its maximum by increasing shear strain, and subsequently, the material ruptured. The values of shear stress were obtained as 255, 285, and 318 kPa for the cement-treated soil specimen and 245, 280, and 318 kPa for the nanocement-stabilized soil specimen under related stresses of 50, 100, and 150 kPa, respectively.

The shear stress–strain curves were compared before and after the nanomaterials were added. The soil hardened, indicating that changing and replacing a portion of the soil with nanocement will have a major effect on the stress–strain behavior of the soil. The shear strain at the highest peak will cause these changes. The effect of nanocement suspension was compared in the shear stress–strain curves. The shear strength and hardness of nanocement-stabilized soil specimens were substantially greater than those of soil. The increase in adhesion and friction angle changes is caused by the nanoparticles activating the cementation function in the treated soil, depositing nanoparticles within the clay pores and enhancing the adhesion between clay particles.

This increase in the proportion of nanocement suspension increases the amount of nanocement in soil cavities and particles, limiting the porosity of the specimens and establishing a link between soil particles. In addition, it increases the maximum stress; by increasing specimen resistance due to further enclosing it, the specimen is forced to resist more during loading owing to high shear stress.

### Image analysis

#### XRF analysis

X-ray fluorescence (XRF) tests were conducted to ensure that the soil was not contaminated with the wear of metal and ceramic balls during the nanomanufacturing process. Tables 3 and 4 present the results of XRF analysis before the nanomanufacturing process. According to Tables 3 and 4, no additional material was added to cement during the nanomanufacturing process.

**Table 3:** XRF analysis for cement before the nanomanufacturing process.

Chemical composition	MgO	Al_2_O_3_	SiO_2_	SO_3_	Na_2_O	K_2_O	CaO	Fe_2_O_3_
Weight (%)	1.59	5.04	21.13	2.30	0.42	0.69	63.30	3.76

**Table 4:** XRF analysis of cement after the nanomanufacturing process.

Chemical composition	MgO	Al_2_O_3_	SiO_2_	SO_3_	Na_2_O	K_2_O	CaO	Fe_2_O_3_
Weight (%)	1.43	5.46	20.28	2.04	0.37	0.49	64.79	4.07

Table 4 shows the results of the XRF test after the nanomanufacturing process.

The XRF test results on the additive before and after the nanomanufacturing process [Tables 3 and 4] revealed slight variations in the cement and nanocement constituent elements.

#### SEM analysis

**Figure 14:**
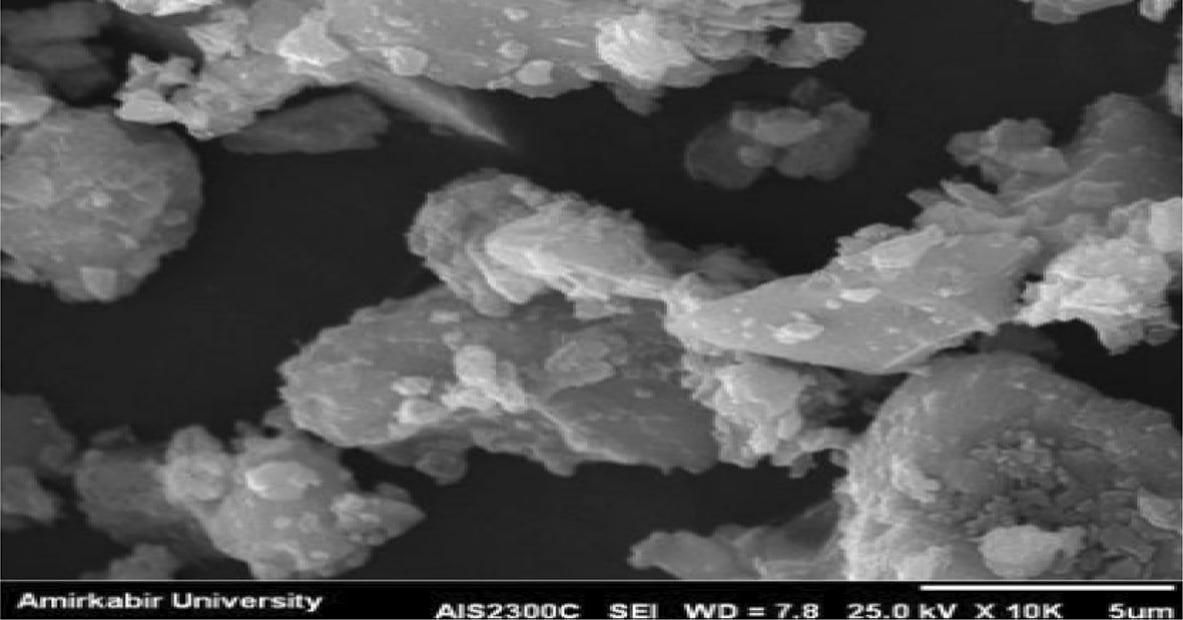
SEM image of cement before the nanomanufacturing process.

**Figure 15:**
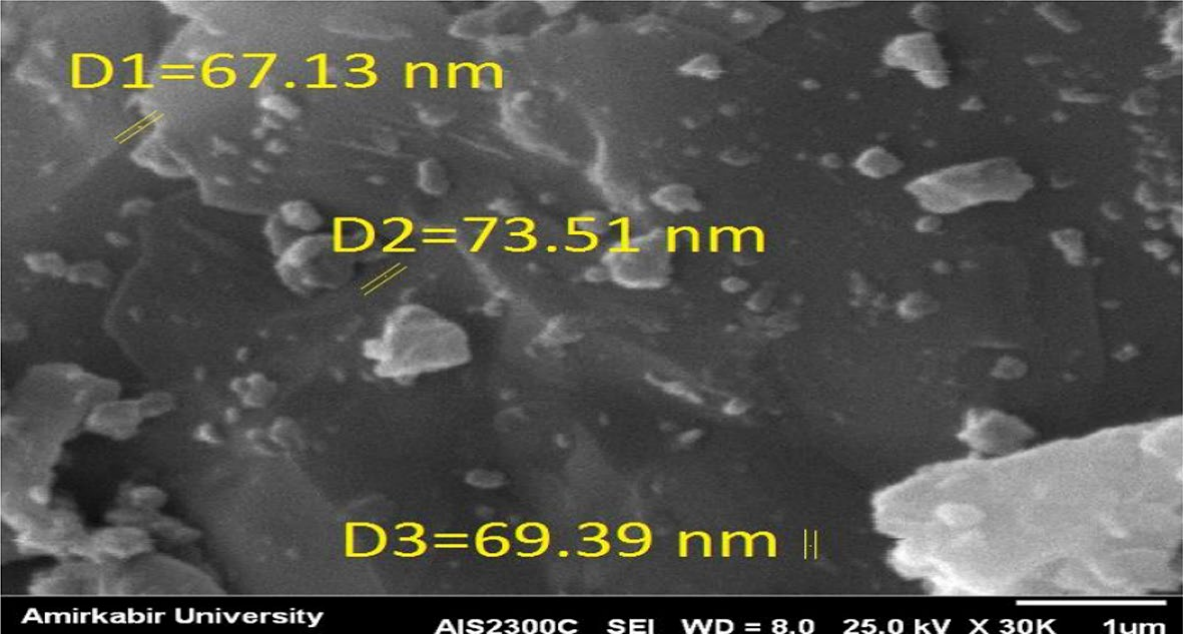
SEM image of cement after the nanomanufacturing process.

A scanning electron microscopy (SEM) is a type of electron microscope that generates images by scanning the surface of a specimen with a concentrated beam of electrons. Figure 14 shows the SEM image of nanocement before the nanomanufacturing process, while Fig. 15 shows the SEM image of nanocement 150 min after the nanomanufacturing process. According to the results, the particle size range was determined between 5 and 56 nm for the SEM image.

#### XRD analysis

**Figure 16:**
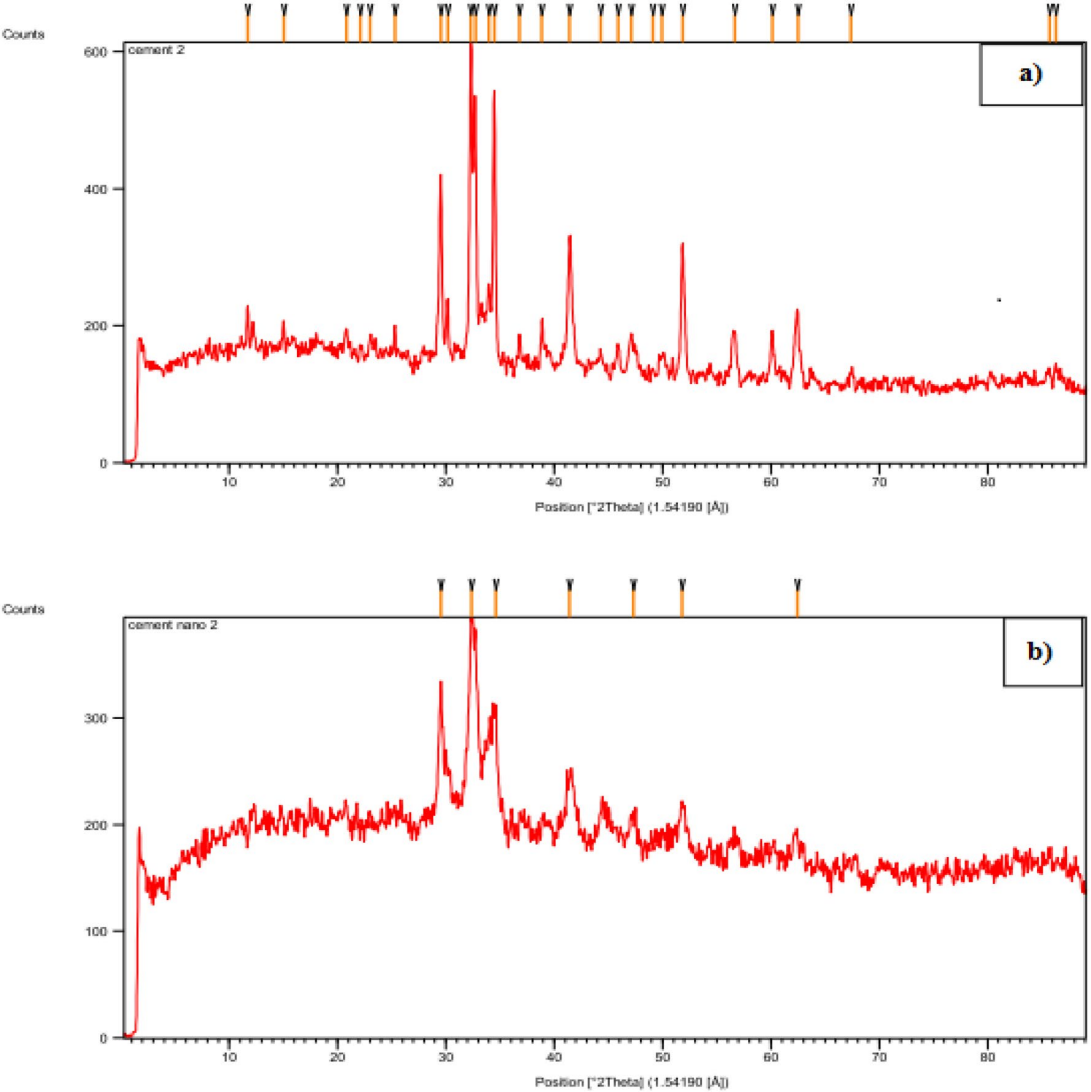
(**a**) XRD diagram of cement before the nanomanufacturing process and (**b**) XRD diagram of cement after the nanomanufacturing process.

X-ray powder diffraction (XRD) analysis was conducted to confirm that the soil was not contaminated by the wear of metal and ceramic balls during nanomanufacturing. Figure 16a,b show the results of the XRD test conducted on 2 g of cement before and after the nanomanufacturing process, respectively.

According to the results presented in Fig. 16a,b, the majority of the peaks in each soil specimen occur at a specific angle and the location of the peaks (representing the mineralogy of the constituent elements) is consistent across all soil specimens, indicating that no other substance was added to the cement.

## Conclusions

This research paper presented the geotechnical investigation of Kelachay clay treated with 0%, 1%, 3%, 5%, and 7% micro- and nanosized cement. A curing time of up to 90 days was evaluated. The behavior of compressive and shear strengths and variations in the Atterberg limits of Kelachay clay soil were investigated. Compressive strength characteristics, including unconfined compressive strength and elastic modulus, were obtained from the unconfined compression test, while shear strength values were obtained from the direct shear test. Subsequently, the results were controlled by the regulatory values of soil structures, and a summary of the outcomes of these tests is provided below.

1. The addition of 7% cement and nanocement during a 90-day curing period increases the unconfined compressive strength of soil specimens treated with nanocement by up to 29 times in comparison to the untreated specimen.
2. The addition of various percentages of nanocement reduces the strain at the rupture of the treated specimens relative to the untreated specimen. In the 90-day specimens treated with a 7% nanocement suspension, there is a 74% decrease compared to the untreated soil.
3. The maximum shear stress of soil stabilized with 7% nanocement suspension in all-round stresses of 50, 100, and 150 kPa was 255, 285, and 340 kPa, and for soil stabilized with cement, it was 250, 281, and 318 kPa, respectively.
4. The addition of various percentages of nanocement causes an increase in the elastic modulus of soil treated with various percentages of nanocement compared to untreated soil at various curing durations.
5. Nano-cement reacts with water in the soil and forms a gel-like substance called C–S–H. This substance fills the gaps between the soil particles and makes them stick together. Nano-cement also helps to create more C–S–H by acting as a nucleus for its growth. This enhances the soil–cement mixture's durability and strength.

This research indicated that nano-cement improved the soil–cement mixture by forming a stronger and denser matrix. Therefore, nano-cement can be used as an effective additive for soil stabilization and improvement.

This research is an initial step toward alternative nanoadditives in geotechnical engineering. Nanoadditives can be used for soil stabilization purposes. The micro- and nanoadditive dosage and its performance depend on soil properties. Soil properties play a critical role in nanosoil improvement techniques. According to this research report, authors recommend further evaluations of various micro- and nanosized additives with untreated soil types in laboratory and full scale studies.

## Data Availability

The datasets generated during and/or analysed during the current study are available from the corresponding author on reasonable request.
